# Introduction to the special series: using evidence to enhance health services for individuals using drugs in rural communities

**DOI:** 10.1186/s13722-024-00489-z

**Published:** 2024-08-23

**Authors:** Erin L. Winstanley, Sterling M. McPherson, P. Todd Korthuis

**Affiliations:** 1 Division of General Internal Medicine, School of Medicine, University of Pittsburgh, 230 McKee Place, Pittsburgh, PA 15213 USA; 2 Elson S. Floyd College of Medicine, Washington State University, Spokane, WA USA; 3 Analytics and PsychoPharmacology Laboratory (APPL), Spokane, WA USA; 4 Program of Excellence in Addiction Research (PEAR), Washington State University, Spokane, WA USA; 5 Oregon Health and Science University, Portland, OR USA

Overdose death rates were higher among rural counties in the United States (U.S.) from 2007 to 2015, after which point the overdose death rate has been higher in urban counties [1]. Nevertheless, many rural communities have been disproportionately impacted by the ongoing overdose epidemic [2]. A recent report, with data collected in 2021–2022 from key stakeholders in rural communities, found that ‘addiction’ and ‘drug and alcohol use’ were ranked within the top 10 Healthy People 2030 priorities [3]. Promising data suggests that overdose fatalities may be declining in some rural areas like West Virginia [4]. Rural communities, however, continue to experience limited resources to adequately address the ongoing demand for services for individuals who use drugs and for those with substance use disorders (SUDs).

Rural communities often lack the health care infrastructure that exists in many large urban areas, that can easily be scaled-up as demand increases, and rural communities have even more limited capacity to scale-up specialty services. Treatment for individuals with SUDs in rural areas often falls on existing primary care providers and hospitals; however, as population density decreases so does availability of virtually all health care facilities. Rural health care providers have been squeezed tight by chronic staffing shortages [5], which have worsened since the onset of the COVID-19 pandemic and the continued closures of rural hospitals [6]. A recent report from the Center for Healthcare Quality and Payment Reform documents that 189 rural hospitals have closed since 2005, and estimates that an additional 200 rural hospital are at imminent risk of closure due to severe financial problems [7]. Barriers to health care access in rural communities include financial constraints, stigma, limited resources (including clinicians), transportation and internet access [8].

The articles included in this special series entitled “Substance Use Care in Rural Communities” showcases empirical research to improve health outcomes for individuals using drugs in rural communities. Several of these studies were funded through the National Institutes of Health’s (NIH) Helping to End Addiction Long-term (HEAL) Initiative^®^ and the National Institute on Drug Drug Abuse’s  (NIDA) Clinical Trials Network (CTN). The CTN was developed 24 years ago to decrease the gap between research and practice [9], with the long-term goal of improving outcomes for individuals with SUDs. To address the gap in research on SUDs in rural communities, the CTN funded five new nodes (Appalachian, Great Lakes, Greater Intermountain, Southern California and Southwest) in 2019. These funding initiatives, along with others like the State Opioid Response (SOR) funding from the Substance Abuse and Mental Health Services Administration (SAMHSA), have been critical to finding solutions in the hardest hit rural communities. Jenkins et al. describe the Rural Opioid Initiative (ROI), a multisite research collaborative designed to advance our knowledge of drug use in rural communities [10]. The ROI cohort includes 3,084 individuals who use drugs in rural communities. The majority of participants (74%) used methamphetamines in the past 30 days. Less than half reported ever receiving medications for opioid use disorder (MOUD) [10]. Qualitative interviews found that participants frequently avoided contacting emergency response services if they witnessed an overdose [10]. This is consistent with existing evidence of the intersection of health disparities (e.g., poverty, lack of healthcare access) and stigma that people with SUDs experience in rural communities, and it points to a need for continued policy and treatment research. Stigma and health disparities was a consistent theme across many of the articles in this special issue, as was less access to evidence-based services (e.g., buprenorphine, syringe service programs). The articles propose multiple solutions to rural challenges such as using technology to remediate transportation barriers, improve clinical workflows, treatment processes and to expand clinician training.

Walters et al. highlighted the hardships that individuals using drugs faced during the COVID-19 pandemic such as food insecurity, job loss and being restricted from accessing public services (e.g., transportation, free Wi-Fi) [11]. Individuals residing in rural communities have higher rates of mortality and morbidity [12] which reflect complex financial, social and chronic comorbid health problems. Health care may not be at the forefront of an individual’s mind when they are struggling with more basic needs like shelter, food and safety. Provision of employment, legal and food assistance may not be seen as within the purview of health care organizations; however, within the hierarchy of needs, patients may require that their basic needs are met before they can engage in non-acute prevention and treatment services. Some rural communities have inadequate or unreliable access to broadband and rely on accessing Wi-Fi at public locations (e.g., libraries, convenience stores if available), which may prevent health care organizations from utilizing telemedicine. And to that point, Burton et al. reported that their intervention to improve Hepatitis C treatment in rural veterans with SUDs could not utilize telemedicine during the pandemic [13]. This was problematic during the peak of the COVID-19 pandemic due to restrictions on in-person interactions and rural communities may want to modify their emergency response plan to enhance Wi-Fi if traditional physical locations with free Wi-Fi are closed or otherwise inaccessible. Pasman et al. also reported transportation barriers and explained that ride sharing services like Uber and Lyft, while commonly available in urban areas, were problematic in rural areas because low demand led to price surges and inconsistent availability [14]. Conversely, they found that greater social support was associated with fewer barriers to methadone access [14]. In rural areas, individuals may rely on their social networks to overcome transportation barriers in the absence of ride sharing services or public transportation systems.

Technological tools are critical to support expanded service access in rural communities and to improve clinician workflow. For example, Smith et al. reports development of the Automated Reinforcement Management System (ARMS), a patient-facing hybrid mobile/web-based system to deliver contingency management (CM) incentives for patients with alcohol use disorder who demonstrate negative alcohol breathalyzer tests using the system [15]. McNeely et al. found that technology was critical to three federally qualified health centers (FQHCs) in Maine successfully screening for tobacco, alcohol and drug use in 93% of patients presenting for an annual health visit [16]. The screening tool was self-administered by patients on a tablet and the data was electronically integrated into the EHR; further, within the EHR system a clinical reminder was built along with a template to guide a brief intervention [16]. Telemedicine approaches have also been proposed as a solution for improving access to buprenorphine in rural communities [17–19]. Hser et al. report barriers to implementing telemedicine-delivered buprenorphine services in a multi-site rural feasibility study conducted during the COVID-19 pandemic [20]. Challenges to implementing telemedicine buprenorphine may have included structural barriers during the COVID pandemic and a study design choice that relied on rural clinics to refer their patients to an outside telemedicine provider [20]. Qualitative research on the acceptability of a mHealth intervention in rural Appalachia found that patients expressed a preference for a personal connection or local health coach [21] and even when conducting research in rural areas, involving rural community members or hiring local research staff can improve study recruitment [22]. While there is evidence that rural residents are very interested in technology-based services [23] and that rural health care providers were able to effectively deliver telemedicine services during the COVID-19 pandemic [24], there remains a need to better understand how to tailor telemedicine and mHealth interventions to optimize engagement and retention of rural patients.

Two articles in the supplement looked at expanding access to buprenorphine, one using financial incentives to encourage adoption at the clinician-level [25] and the second using a learning collaborative to facilitate buprenorphine treatment at the organizational-level [26]. Efforts to expand evidence-based services in rural communities may be hampered by health care provider stigma towards individuals who use drugs. Haggerty et al. found that many clinicians (44%) that were not waivered to prescribe buprenorphine, prior to the removal of the DEA waiver, were not interested in buprenorphine training even if they were financially compensated for their time [25]. Further, they found that this reluctance appeared greater in physicians who endorsed stigmatizing attitudes or beliefs about patients with SUDs [25]. This work illuminates how deeply entrenched these stigmatized views are among some rural health care providers and that applying financial incentives to accelerate adoption, may not work as well in rural areas. Although provider stigma towards patients with opioid use disorders is highly prevalent and ubiquitous [27], it is possible that financial incentives would also be ineffective in urban providers. Murray et al. used an ECHO learning collaborative to improve implementation of evidence-based services, which was successful in making smaller incremental advances using quality improvement strategies and it seemed to be most effective in increasing the number of patients treated in low volume clinics [26]. Other teams have also reported success with this approach [28, 29], which represent encouraging developments and an approach that has the potential for replication in other rural communities.

The COVID-19 pandemic has had positive unintended consequences for the delivery for addiction treatment. Levander et al. found that rural opioid treatment programs (OTPs) were willing to loosen their stringent or “high threshold-to-receive” services resulting in some patients feeling more empowered in their recovery [30]. Indeed, Amram et al. also reported that the natural experiment of allowing take-home methadone more broadly during the COVID-19 pandemic resulted in no change in illicit opioid use [31]. This is a finding that could represent a new path for more liberal use of take-home methadone that could benefit rural-dwelling people in the United States (US), given that distance from a methadone clinic impacts patients’ ability to regularly attend [32]. The NIDA CTN has funded a new study (CTN-0131) that is a hybrid effectiveness implementation trial of office-based methadone that will generate empirical data needed to better understand the feasibility of this approach in the US. Nguyen et al. reported on clinician characteristics associated with the provision of methadone in primary care practices in rural Vietnam [33]. They found that clinicians that held less stigmatizing views of patients who used drugs and had some knowledge or experience of methadone treatment were more confident in delivering methadone treatment [33]. Australia, Canada and the United Kingdom offer methadone services outside of the specialty addiction treatment system and if ever adopted in the US, it has the potential to significantly increase MOUD in rural areas.

Rural communities across the US also have lower access to syringe service programs (SSPs) [34] that are effective for reducing risk of hepatitis C (HCV) and HIV transmission [35]. Romo et al. documented that individuals in rural New England with limited access to SSPs were more likely to rely on either purchasing syringes at local pharmacies or secondary access through their social network [36]. Secondary access to syringes was not associated with risky injection practices [36]. Gupta et al. identified mental health issues (particularly post-traumatic stress disorder) as a potential driver of risky needle sharing, and mental health issues were associated with increased risk of HCV in a study conducted in northern New Mexico [37]. The authors recommended an expansion of mental health services as a potential intervention for reducing risky injection drug use and HCV transmission [37]. Burton et al. piloted an intervention using motivational interviewing and CM to improve direct acting antiviral medication adherence and treatment completion among rural veterans with SUDs [13]. The majority of the patients that participated in the program continued to use drugs and nevertheless, 19 of 20 achieved a sustained virologic response [13]. CM is also one of the only evidence-based interventions demonstrated to reduce methamphetamine use [38]; unfortunately widespread adoption of CM has been thwarted due to federal anti-kickback regulations [39]. Fortunately, some states have recently obtained Medicaid waivers and are now able to begin to rollout CM programs [40].

Rural communities may have lower rates of adoption of evidence-based practices, perhaps because of healthy skepticism of their effectiveness given that rural communities are not routinely included in the research. Watson et al. found that rural doctors, compared to their urban counterparts, were reluctant to refer to peer recovery specialists [41]. It was beyond the scope of their study to investigate the causes of this reluctance, but one might hypothesize that rural clinicians may be less inclined to make referrals to novel services (in this case, peer recovery specialists) in the absence of a personal connection or first-hand knowledge of the efficacy of the intervention. In resource-scarce environments, early adoption [42] may be perceived as too risky with unknown economic or social consequences. Efforts to expand implementation and adoption of novel services that improve outcomes for patients with SUDs may need to tailor their approach to highlight participation of rural communities in these studies, whenever possible, and to integrate rural clinicians’ experiences integrating these services into their local practices.

The articles in this supplement provide some clues on how to foster resilience, particularly with respect to social support for individuals with SUDs. In general, social support is a key ingredient to good health [43] and multiple theories of health seeking behavior include social support as an enabling characteristic [44]. Pasman et al. investigated barriers to retention in methadone treatment and to overcome these barriers they recommended (1) allowing more flexible methadone regulations, (2) improving co-location or care coordination and (3) expanding peer and family supports [14]. Importantly, they also found that patients with worse mental health symptoms perceived greater access barriers [14]. It is easy to imagine that mental health symptoms, depression in particular, causes lower self-efficacy which is needed to overcome service utilization barriers [14]. Hence, the successful integration of interventions in rural communities may need to include components that actively improve self-efficacy at both the organizational- and individual-levels. For example, peer recovery support specialists in rural communities could serve as a much-needed bridge between non-treatment seeking populations and mental health and substance use treatment services [45, 46].

Many of the challenges faced by rural communities in combatting the overdose epidemic such as the scarcity of services, limited resources, health care professional shortages and transportation barriers are not unique to the US as similar challenges exist in rural communities around the globe [47]. These challenges often seem insurmountable as they would require improvements in rural communities’ economic, political and social systems. Nevertheless, we can look beyond the geographic boundaries that define our own communities to find creative and novel solutions to improve health outcomes for individuals with SUDs. These solutions, some of which are described in this supplement, often entail stepping outside traditional service settings and tailoring evidence-based interventions to the local community. Strategies to build resilience may include bolstering social capital and using local connections, such as peer recovery specialists, to improve access to prevention and treatment services. If we want to find solutions to the rural overdose crisis, undoubtedly we need to continue to expand funding opportunities and improve the inclusion of individuals in rural communities in clinical research.
